# Supplementary material for: Complexome profiling of the Chlamydomonas psb28 mutant reveals TEF5 as an early PSII assembly factor
Source: Plant Cell. 2025 Mar 17;37(6):koaf055. doi: 10.1093/plcell/koaf055 (PMC12164590; doi:10.1093/plcell/koaf055)
Supplement: koaf055_Supplementary_Data [file koaf055_supplementary_data.zip › psb28_interactive.zip!/plots/intensity/qualityPlots/peptides/PDH2_Cre03.g194200.t1.2.html]

### Cre03.g194200.t1.2

Trivial name: PDH2  
SeqCoverage [median]: 11.60  
Mass: 40.25 kDa  
  
 

| Sequence | MissedCleavage | Mass [Da] | wtIntensity | muIntensity | Score |
| --- | --- | --- | --- | --- | --- |
| EGTDVSIFTYSR | 0 | 1373.65 | 5345.18 | 1962.42 | 91.86 |
| EIMMWEALR | 0 | 1177.56 | 387.14 | 1179.78 | 62.00 |
| IVGATAPVRA | 1 | 953.57 | 9381.42 | 8162.61 | 105.57 |
| YVVMQAVSELVK | 0 | 1364.74 | 599.56 | 713.72 | 105.20 |
|  |  |  | 15713.3 | 12018.5 |  |
